# Prediction of motor and non-motor Parkinson’s disease symptoms using serum lipidomics and machine learning: a 2-year study

**DOI:** 10.1038/s41531-024-00741-y

**Published:** 2024-06-25

**Authors:** Jasmin Galper, Giorgia Mori, Gordon McDonald, Diba Ahmadi Rastegar, Russell Pickford, Simon J. G. Lewis, Glenda M. Halliday, Woojin S. Kim, Nicolas Dzamko

**Affiliations:** 1 Brain and Mind Centre and Faculty of Medicine and Health, School of Medical Sciences, University of Sydney, Camperdown, NSW 2050 Australia; 2 Sydney Informatics Hub, University of Sydney, Camperdown, NSW 2050 Australia; 3 Bioanalytical Mass Spectrometry Facility, University of New South Wales, Sydney, NSW 2052 Australia

**Keywords:** Parkinson's disease, Predictive markers, Metabolomics, Translational research

## Abstract

Identifying biological factors which contribute to the clinical progression of heterogeneous motor and non-motor phenotypes in Parkinson’s disease may help to better understand the disease process. Several lipid-related genetic risk factors for Parkinson’s disease have been identified, and the serum lipid signature of Parkinson’s disease patients is significantly distinguishable from controls. However, the extent to which lipid profiles are associated with clinical outcomes remains unclear. Untargeted high-performance liquid chromatography-tandem mass spectrometry identified >900 serum lipids in Parkinson’s disease subjects at baseline (*n* = 122), and the potential for machine learning models using these lipids to predict motor and non-motor clinical scores after 2 years (*n* = 67) was assessed. Machine learning models performed best when baseline serum lipids were used to predict the 2-year future Unified Parkinson’s disease rating scale part three (UPDRS III) and Geriatric Depression Scale scores (both normalised root mean square error = 0.7). Feature analysis of machine learning models indicated that species of lysophosphatidylethanolamine, phosphatidylcholine, platelet-activating factor, sphingomyelin, diacylglycerol and triacylglycerol were top predictors of both motor and non-motor scores. Serum lipids were overall more important predictors of clinical outcomes than subject sex, age and mutation status of the Parkinson’s disease risk gene *LRRK2*. Furthermore, lipids were found to better predict clinical scales than a panel of 27 serum cytokines previously measured in this cohort (The Michael J. Fox Foundation LRRK2 Clinical Cohort Consortium). These results suggest that lipid changes may be associated with clinical phenotypes in Parkinson’s disease.

## Introduction

Parkinson’s disease (PD) is a neurodegenerative condition that currently has no disease-modifying treatments. A major hurdle for novel treatment development and testing is the limited understanding of factors which contribute to a likely multifactorial disease process, and the associated heterogenous clinical phenotypes. The cause of PD is unknown, however, large-scale sequencing studies have compellingly identified several risk genes that have a lipid-related function, including *GBA1*, *GALC*, *SMPD1*, *SREBF1*, *ELOVL7*, *DGKQ*, *ASAH1* and *PLA2G6*^1–10^, with *GBA1* mutations being the strongest genetic risk factor for PD. In addition to their critical structural roles, many lipids act as signalling molecules in diverse pathways including cell death and other integral cell processes such as mitochondrial function and glucose/insulin signalling^11–13^. Further, α-synuclein aggregation is facilitated by interactions with sphingolipid^14^ and phospholipids^15,16^ (reviewed in ref. ^17^). Intriguingly, a large-scale study of type 2 diabetes patients found that the use of glucagon-like peptide-1 (GLP-1) receptor agonists and inhibitors of the GLP-1 degrading enzyme dipeptidyl peptidase 4 (DPP-4) significantly reduced the incidence of PD. Meta-analyses also indicate an overall protective effect of statin use^18–20^. Both statins, GLP-1 agonists and DPP-4 inhibitors affect cholesterol and triacylglycerol levels^21–24^. Therefore, with increasingly diverse roles becoming more apparent for lipids, their study offers new prospects to understand neurodegenerative disease.

Despite the evidence indicating that lipids may play a role in PD, the predictive value that baseline blood lipids may have for both motor and non-motor scales has not been explored longitudinally, although plasma lipids estimating cognitive and motor severity scores have been assessed cross-sectionally^25^. Two previous studies have assessed baseline blood lipids for predicting a binary clinical outcome, namely, the future diagnosis of dementia^26^ or PD^27^ and have not investigated the degree to which lipids may predict motor and non-motor clinical scale scores, which are used as tools for assessing clinical trial drug effectiveness. One study of *n* = 43 PD subjects found that a panel of baseline serum lipids could discriminate between those that progressed to dementia and those that remained cognitively stable after 3 years^26^. Another study of *n* = 39 each of PD and control subjects found that baseline plasma metabolites, which included lipids, contributed to the prediction of PD incidence after 15 years^27^. Given the range of motor and non-motor symptoms in Parkinson’s disease, comparatively assessing the ability of baseline serum lipids to predict future scale scores of olfaction, depression, motor function and quality of life in PD subjects could be valuable.

In an untargeted investigation of >1000 lipids in serum from two cohorts (*n* = 221 and *n* = 315), we previously reported that the serum lipid signature of PD patients was significantly distinguishable from those without PD^28^. The atypical lipid profiles implicated sphingolipid metabolism, insulin signalling and mitochondrial function as major metabolic pathways dysregulated in PD. A proportion of this cohort was followed longitudinally, and nine motor and non-motor clinical severity scales were collected over 2 years. To what extent baseline serum lipids levels are predictive of motor and non-motor clinical severity scales after 2 years is investigated in this study. Given that inflammatory markers have been extensively studied in PD^29^ and are implicated in the prediction of PD severity^30^, the predictive performance of 27 serum cytokine levels previously published for this cohort is also compared to serum lipids^30^. Machine learning was used to train a statistical model that could make predictions of unknown clinical data using baseline serum markers. This untargeted investigation has provided lipid candidates predicting future motor and non-motor PD progression scores, and found them to generally supersede the predictive ability of serum cytokines. These results may guide the future investigation of the role of lipids in PD progression.

## Results

### Evaluation of longitudinal clinical data

Demographic and clinical data of PD subjects at baseline and 2-years follow-up are shown in Table 1. Repeated measures univariate analysis indicated that Geriatric Depression Scale, Schwab and England Activities of Daily Living Scale, University of Pennsylvania Smell Identification Test, Movement Disorder Society Unified Parkinson’s Disease Rating Scale part three (UPDRS III) and Hoehn and Yahr scores were statistically significantly higher from baseline after 2 years (all *p* < 0.05, Table 1). Montreal Cognitive Assessment (MoCA), Epworth Sleep Scale, Scales for Outcomes in Parkinson’s Disease-Autonomic dysfunction (SCOPA-AUT) and Rapid Eye Movement (REM) Sleep Disorder Questionnaire (RBDSQ) scores did not statistically significantly change over the 2-year period, so they were not studied further (all *p* > 0.05, Table 1).

**Table 1:** Demographic and clinical data for the PD participants in the LRRK2 Ashkenazi Jewish cohort

	Baseline	+2 years	2 years - baseline
*N*	122	67	
Age	70.4 ± 0.8 (49–90)	71.1 ± 1 (50–89)	
Sex (M/F)	75/47	46/21	
Age at diagnosis	58.4 ± 0.9 (34–83)	58.5 ± 1.2 (34–83)	
LRRK2 G2019S (N/Y)	57/61	34/29	
GBA1 (N/Y)	94/3	48/2	
LRRK2/GBA1 (N/Y)	93/4	63/4	
LED	560.3 ± 38.5 (0–2280)	707.6 ± 56.1 (0–2600)*	
MoCA	25.1 ± 0.4 (5–30)	25.15 ± 0.5 (10–30)	−0.8 ± 0.4
UPDRS III	22.7 ± 1.2 (2–67)	25.4 ± 1.7* (1–71)	3.6 ± 1.1
Epworth Sleep	8.7 ± 0.4 (0–22)	9.4 ± 0.6 (1–24)	0.2 ± 0.6
Depression	4.2 ± 0.4 (0–14)	4.5 ± 0.5* (0–15)	0.9 ± 0.4
Activities of Daily Living	76. 7 ± 2 (10–100)	72.9 ± 2.4** (20–100)	−7.5 ± 2
SCOPA-AUT	22.6 ± 1.2 (0–62)	24.6 ± 1.5 (4–52)	1.7 ± 1.8
REM sleep	3.9 ± 0.3 (0–12)	3.7 ± 0.4 (0–12)	−0.5 ± 0.3
UPSIT	20.8 ± 0.9 (2–39)	17.5 ± 1* (7–39)	−1.3 ± 0.8
Hoehn & Yahr	2.4 ± 0.1 (0–5)	2.7 ± 0.1* (1–5)	0.5 ± 0.1
NfL	126.6 ± 30.8 pg/ml		
α-synuclein	17.9 ± 2.3 ng/ml		

Demographic data of PD patients only at baseline, 1 year and 2 years from the Michael J. Fox Foundation LRRK2 Clinical Cohort Consortium. Geriatric Depression Scale, Hoehn and Yahr, Schwab and England Activities of Daily Living Scale, UPSIT and UPDRS III significantly worsened over the 2-year period. A comparison of motor and non-motor clinical variables across time points was performed with a repeated measured ANOVA except for Hoehn & Yahr, which was analysed via Kruskal Wallis and Mann-Whitney U tests. Values are mean ± SEM (range). Demographics were not compared between time points. LRRK2 mutation carriers were all G2019S, while GBA1 carriers were N409S or L29Afs.

*LED* Levodopa Equivalent Dose, *MoCA* Montreal Cognitive Assessment, *UPDRS III* Unified Parkinson’s Disease Rating Scale part 3, *Depression* Geriatric Depression Scale, *Activities of Daily Living* Schwab and England Activities of Daily Living Scale, *SCOPA-AUT* Scales for Outcomes in Parkinson’s Disease-Autonomic dysfunction, *UPSIT* University of Pennsylvania Smell Identification Test, *NfL* neurofilament light.

*p < 0.05 compared to baseline; **p < 0.001 compared to baseline.

### Machine learning to predict longitudinal clinical outcomes

**Fig. 1:**
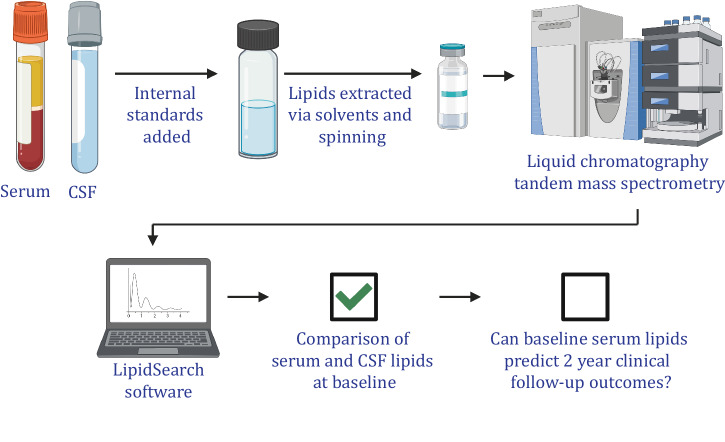
Method overview. Simplified workflow of untargeted lipidomics using serum and CSF which was previously published^28^ (except for the last step, which is the current publication pertaining to longitudinal clinical data). Created with BioRender.com.

**Fig. 2:**
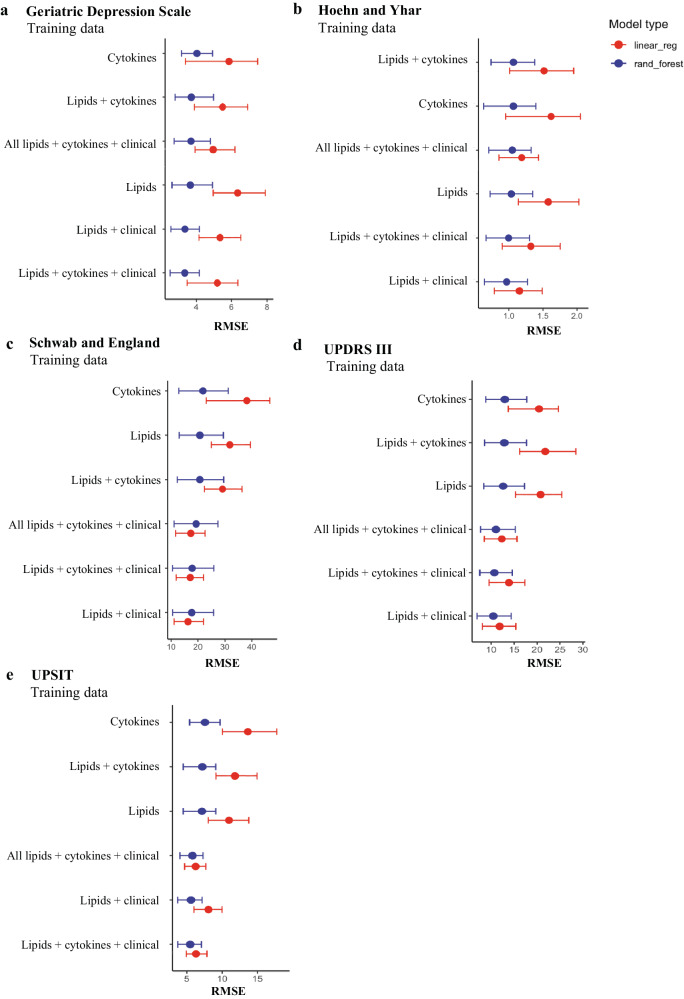
Machine learning models. Comparison of random forest (blue) and elastic net linear regression (red) models to predict the (**a**) Geriatric Depression, (**b**) Hoehn and Yahr, (**c**) The Schwab and England Activities of Daily Living scale, (**d**) Unified Parkinson’s Disease Rating Scale (UPDRS III) and (**e**) University of Pennsylvania Smell Identification Test (UPSIT) scale scores after 2 years in Parkinson’s disease participants. The predictors used are combinations of baseline lipids and cytokines with and without baseline clinical data. ‘All’ lipids is the entire lipid dataset whereas ‘lipids’ is the subset of lipids that separate outcomes from the OPLS. The analysis was performed on the training set (*n* = 54). RMSE root mean square error.

An overview of the methods which generated the lipidomics data is summarised in Fig. 1. Using RMSE as a readout of model performance, random forest analysis of serum lipid and cytokine markers generally performed better than linear regression elastic net models to predict the Geriatric Depression Scale, Hoehn and Yahr, Schwab and England Activities of Daily Living Scale, University of Pennsylvania Smell Identification Test (UPSIT) and UPDRS III (Fig. 2) scores over the 2-year period. Random forest analysis was therefore used to compare models which used baseline clinical and demographic data in combination with either lipids, cytokines or lipids and cytokines. In addition, the model performance using all available lipids was compared to a selection of lipids generated from OPLS-DA as outlined in the methods.

**Fig. 3:**
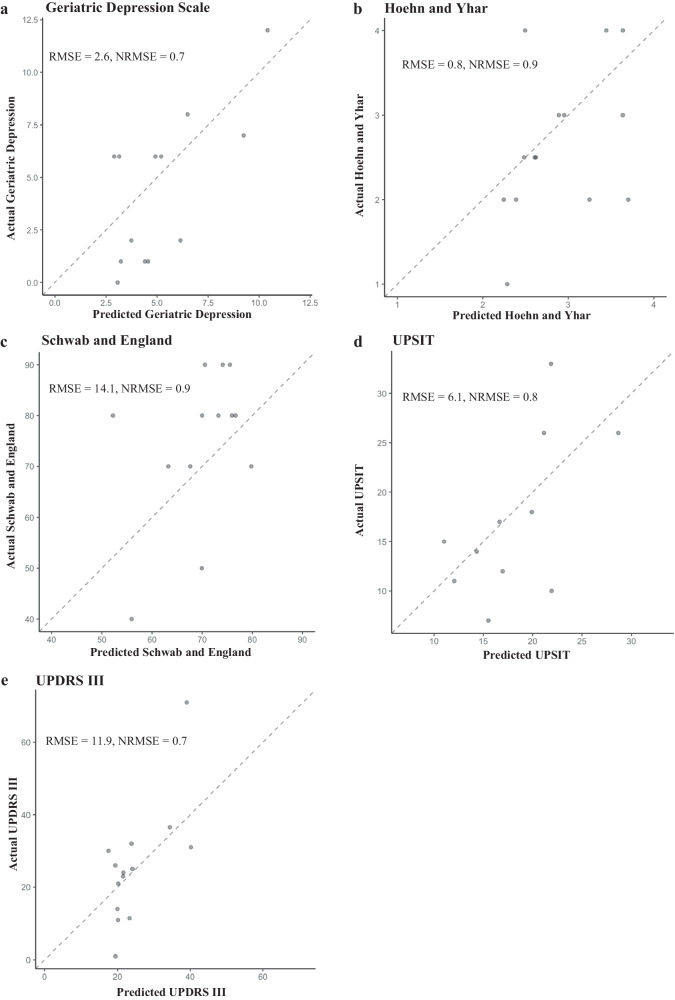
Random forest prediction of longitudinal clinical outcomes. R2 plots of the random forest model using selected lipids plus baseline clinical and demographic data in the testing set for the prediction of (**a**) Geriatric Depression Scale, (**b**) Hoehn and Yahr, (**c**) Schwab and England Activities of Daily Living Scale, (**d**) UPSIT and (**e**) UPDRS III scores in Parkinson’s disease subjects after 2 years. NRMSE normalised root mean square error.

Predictive models with baseline serum lipids alone performed better than cytokines alone, as indicated by lower RMSE scores in the former (Fig. 2). As expected, the addition of clinical and demographic data to models using serum lipids levels improved model performance (lower RMSE scores) when compared to models without clinical and demographic data (Fig. 2). In the training set, the lipids plus clinical and demographic data random forest model performed best for the prediction of the Geriatric Depression Scale, Hoehn and Yahr, Schwab and England Activities of Daily Living Scale (Fig. 2), UPDRS III and UPSIT scores at 2 years (Fig. 2). In the testing set, for the Geriatric Depression Scale, Hoehn and Yahr and UPSIT prediction, the lipids plus clinical and demographic data random forest model (Fig. 3) performed similarly to lipids plus cytokines plus clinical and demographic data. For UPDRS III score prediction, the lipids plus clinical and demographic data random forest model performed best (Fig. 3). For the prediction of the Schwab and England Activities of Daily Living Scale scores after 2 years, the lipids plus clinical and demographic data model (Fig. 3) had a similarly low RMSE score to all lipids alone. The inclusion of additional serum biomarkers NfL and α-synuclein to the serum lipids plus clinical and demographic data models made no improvements to the RMSE for any of the clinical scale prediction models (Supplementary Table S2). The serum lipids plus clinical and demographic data model performance is therefore shown in Fig. 3. Using NRMSE as a comparative measure of model performance, the best-performing prediction models were for the Geriatric Depression Scale and UPDRS III (both NRMSE = 0.7, Fig. 3).

### Top variables contributing to prediction models

The top 10 variables contributing to the serum lipids plus clinical and demographic data random forest model of the Geriatric Depression Scale, Schwab and England Activities of Daily Living Scale, UPSIT and UPDRS III score prediction are presented in Table 2 and the full list is presented in Supplementary Table S3. For Hoehn and Yahr prediction, other than Hoehn and Yahr baseline score and PC 32:1e, variables contributed similarly to prediction, suggesting that specific individual lipid variables were not strong predictors of Hoehn and Yahr in this cohort (Supplementary Table S3). Interestingly, lipids were generally more important to the prediction of motor and non-motor clinical scores than the presence of the LRRK2 G2019S mutation, sex and age (Table 2 and Supplementary Table S3).

**Table 2:** Top predictors of clinical data scores

Variable	Importance	Variable	Importance
Geriatric Depression Scale		Schwab and England Activities of Daily Living	
Geriatric Depression baseline	293.9	Schwab and England baseline	5846.4
LPE 16:0	44.5	PC 32:1e	1764.1
PE 18:1_20:4	35.1	Age	1321.7
SM d39:2	12.5	LPE 16:0	773.0
LPE 20:3	10.9	PE 16:0_18:1	634.7
SM d40:3	10.8	PAF 12:0e	596.9
LPC 16:0	7.5	DG 16:0_18:3	562.7
PC 33:3	7.5	PAF 18:2e	463.3
PAF 18:2e	7.0	PE 18:1_20:4	388.7
PC 40:6	7.0	PE 18:0_18:1	374.1
UPSIT		UPDRS III	
UPSIT baseline	1459.2	UPDRS III baseline	3319.1
PAF 12:0e	86.7	Age	190.0
PC 39:6	83.3	TG 14:0_18:2_18:3	164.1
PC 38:6e	38.3	PAF 12:0e	163.0
PC 40:6	31.1	PC 33:3	144.6
LPC 20:4	23.7	DG 20:3_18:2	141.5
PC 37:6	22.9	LPE 20:3	134.1
TG 14:0_18:2_18:3	21.3	SM d18:1_17:0	125.5
PC 44:6e	18.3	PC 32:0e	119.8
DG 18:1_18:2	18.3	PC 44:6e	118.2

The top 10 variables at baseline and their importance scores contributing to the prediction of Geriatric Depression Scale, The Schwab and England Activities of Daily Living scale, UPSIT and UPDRS III score at 2 year follow-up from the selected lipids plus clinical and demographic data random forest models. *N* = 122 Parkinson’s disease participants at baseline and *N* = 67 at 2 year follow-up.

*UPDRS III* Unified Parkinson’s Disease Rating Scale, *UPSIT* University of Pennsylvania Smell Identification Test, *DG* diacylglycerol, *LPE* lysophosphatidylethanolamine, *PAF* platelet-activating factor; *PC* phosphatidylcholine, *PE* phosphatidylethanolamine, *SM* sphingomyelin and *TG* triacylglycerol.

Phospholipids including phosphatidylcholine and platelet-activating factor featured in the top 10 contributing lipids to the prediction of Geriatric Depression Scale, Schwab and England Activities of Daily Living Scale, UPSIT and UPDRS III scores after 2 years (Table 2). In addition, lysophosphatidylethanolamine was a top predictor of the Geriatric Depression Scale, Schwab and England Activities of Daily Living Scale and UPDRS III scores (Table 2). Glycerolipids including triacylglycerol were additionally important to the top 10 contributing predictors of UPSIT and UPDRS III scores, while diacylglycerol was a top predictor of UPSIT, UPDRS III and Schwab and England Activities of Daily Living Scale scores (Table 2). Sphingomyelin was important for the prediction of the Geriatric Depression Scale and UPDRS III scores after 2 years (Table 2).

To determine whether the top predictive lipids were associated with use of dopamine medication, participants who were not taking dopamine medication at baseline blood collection were selectively investigated. In these participants (*n* = 8), correlation analyses indicated that the top predictive lipids (Table 2) were not significantly associated with levodopa equivalent daily dosage at 2 years in subjects who were not on levodopa at the time of blood collection (*p* > 0.05 for all correlations, *n* = 8, Supplementary Table S4). To see whether the top predictors may be affected by *GBA1*, top predictors (Table 2) were compared in longitudinally followed *GBA1*+ (*n* = 6) and *GBA1*- (*n* = 42) cases. Univariate analyses covarying for age and sex indicated that the top predictors (Table 2) were not significantly different between *GBA1+* and *GBA1-* cases (all *p* > 0.05, Supplementary Table S5).

## Discussion

To determine if baseline blood markers could predict future clinical outcomes, this study took baseline levels of over 900 serum lipids in PD subjects collected from an untargeted lipidomics investigation and determined their potential for predicting motor and non-motor clinical severity scores after a 2 year longitudinal follow-up. This study builds on a previous publication from this cohort which demonstrated that the baseline serum lipid signature of PD patients is significantly distinguished from those without PD^28^. A proportion of this cohort was longitudinally followed, and the value of baseline serum lipid markers to predict future clinical outcomes was assessed. Phospholipid, sphingolipid and glycerolipid species were the top predictors of both motor and non-motor scales. Phosphatidylcholine and platelet-activating factor species were found to be particularly important for the prediction of the Geriatric Depression Scale, Schwab and England Activities of Daily Living Scale, UPSIT and UPDRS III scores. Sphingomyelin species were a top predictor of the Geriatric Depression Scale and UPDRS III scores, while triacylglycerol and diacylglycerol species were the top predictors for UPSIT and UPDRS III scores. In addition, the predictive performance of baseline serum lipids was compared to that of 27 serum cytokines for motor and non-motor scales and it was found that lipids more accurately predicted the Geriatric Depression Scale, Schwab and England Activities of Daily Living Scale, UPSIT and UPDRS III scores after 2 years from baseline. Further, the predictive performance of models which used baseline serum lipids were not further improved by the addition of baseline levels of serum α-synuclein or neurofilament light. Interestingly, baseline serum lipid levels contributed more to the prediction of motor and non-motor scales than sex, LRRK2 G2019S mutation status and age. The exception was age being a top contributor to UPDRS III prediction. A cross-sectional study found that plasma dihydroglobotriaosylceramide was a stronger estimator of UPDRS III score than age at baseline^25^, however, this lipid was not detected in serum from the longitudinally followed cohort in this untargeted analysis. Future studies are therefore needed to verify that this particular sphingolipid is able to predict UPDRS III scores longitudinally.

This study compared random forest and elastic net machine learning algorithms for the prediction of PD clinical severity scales and found that overall, random forest had the best predictive performance using the baseline serum lipids plus clinical and demographic data model. Using NRMSE as a comparative measure of model performance, the best-performing prediction models were for the Geriatric Depression Scale and UPDRS III. Whether the predictive model for Geriatric Depression Scale has utility outside of a PD context remains to be determined. In addition, how the UPDRS III prediction model performs in other movement disorders which use the UPDRS III is an area of future research. Although the prediction model performed better with serum lipids compared to cytokines, it is noted that the top contributing lipids of motor and non-motor scales include those that have inflammatory functions, such as platelet-activating factor^31^. Platelet-activating factor is often produced in response to triggers such as oxidative stress^31^, and can initiate apoptosis in neurons^32^ and glia^33,34^. Binding of platelet-activating factor to the platelet-activating factor receptor can induce diacylglycerol production^35–38^. Serum diacylglycerol 16:0_18:3 (DG 34:3) was the #7 contributor to Schwab and England Activities of Daily Living Scale score, which is consistent with a recent metabolomics study which found plasma DG 34:3 was a top contributor to PD incidence prediction^27^. This study also found DG 16:0_18:3 (DG 34:3) contributed to other scales (UPDRS III, UPSIT, Geriatric Depression Scale and Hoehn and Yahr) but to a lesser extent (#62 - #78 on variable importance lists). Whether the DG 34:3 found in this study and that found previously represent diacylglycerols with the same fatty acid chain breakdown remains to be determined. Diacylglycerol has several functions, including being important for energy, mitochondrial fission^39^ and glucose/glycogen signalling^40^. Given that plasma and serum comprise lipids from multiple tissues and the diet, the origin of the predictive lipids identified requires further investigation. Diacylglycerol is a precursor to phosphatidylethanolamine and phosphatidylcholine, which have several roles including structural roles in membranes^11^. The top contributing phospholipids lysophosphatidylethanolamine, phosphatidylethanolamine, lysophosphatidylcholine and phosphatidylcholine are also important for phospholipid metabolism [KEGG^41^ pathways map00564]. Interestingly, lysophosphatidylethanolamine 16:0 and lysophosphatidylcholine 16:0, which were top predictors of PD scale scores in this study, have recently been shown to inhibit the aggregation of α-synuclein^42^. Triacylglycerol and diacylglycerol are important for several pathways including oxidative phosphorylation and thermogenesis [KEGG map04714], glycogen production, GLUT4 translocation and glucose uptake [KEGG map04931] and glycerolipid metabolism [KEGG map00561]. Sphingomyelin, which was a top predictor of the Geriatric Depression Scale and UPDRS III scores, can be synthesised from phosphatidylcholine via sphingomyelin synthase^11^. Sphingomyelin is involved in sphingolipid metabolism [KEGG map00600], sphingolipid signalling [KEGG map04071] and necroptosis pathways [KEGG map04217].

To investigate whether dopamine medication was associated with the top predictive lipids, an analysis was performed in a subgroup of participants (*n* = 8) that were unmedicated at baseline blood collection. Levodopa did not significantly correlate with any of the top predictive lipids, suggesting that clinical scores rather than levodopa are associated with the top predictors in this study, however, a larger sample size of drug-naïve PD cases is necessary to confirm this.

Body fat may correlate to some lipids^43^, and this study did not incorporate measures such as body mass index (BMI). However, it has previously been shown that BMI does not contribute to UPDRS prediction^25^. The clinical data scales MoCA, RBDSQ, SCOPA-AUT and Epworth Sleep Scale did not significantly change over a 2-year period in this cohort and were therefore not appropriate for the prediction models. Although Hoehn and Yahr scores increased after 2 years, the narrow range of scores (mean baseline score 2.4 ± 0.1 SEM and 2-year mean score 2.7 ± 0.1) may have precluded the development of an effective prediction model. Those with lower Hoehn and Yahr scores (between 1 and 2.5) have been shown to progress faster than those with higher scores (between 3 and 5)^44^. The current study had subjects which were on average at the border between faster and slower progressing at baseline. Therefore, to capture the fastest progressing PD demographic in future longitudinal studies, it could be strategic to include a large proportion of those at Hoehn and Yahr stage 1–2 at baseline. A comparison of the top predictive lipids between the small sample of *GBA1+* cases available (*n* = 6) and *GBA1-* cases (*n* = 42) showed that the levels of the top predictive lipids from the machine learning models were not significantly different between the two groups. This suggests that mutations in *GBA1* variants are not drivers of the model outcomes, however, a larger sample set of *GBA1* carriers is needed to confirm this. In addition, other genotypes which may affect lipid function, such as *GALC, SMPD1* and *ASAH1*, remain unknown for the current cohort. Despite finding that serum lipids were better predictors of clinical scales than cytokines and in some instances sex, age and mutation status, the prediction model error would need to be addressed before clinical utilisation. It would be interesting to see if this could be improved by controlling for additional genotypes, medication use (such as anti-depressants) and increasing sample size. Assessment of predictor robustness is necessary and a longer follow-up, larger cohort and more diverse clinical scores are advised to predict these scales in the future. In addition, future investigations should validate lipids using authentic standards to confirm specific species, differentiate potential isomers and isobars and identify the fatty acid chain breakdown of unresolved chains. It would also be of interest to measure lipid levels at multiple time points to assess marker stability and potential as a disease progression biomarker. In conclusion, this untargeted study generated a shortlist of serum lipid candidates that may have a role in the progression of PD. Discerning whether combining the top predictive serum lipids with metabolic markers or proteins other than cytokines improves predictive model performance may prove valuable. Finally, it would be of great interest to measure these lipids in pre-clinical PD cohorts such as those with idiopathic REM sleep behaviour disorder^45^.

## Methods

### Patient samples and clinical data

Lipid^28^ and cytokine^30^ data used in this study were collated from two previous investigations using serum samples and matching clinical data obtained from the Michael J. Fox Foundation LRRK2 Cohort Consortium. For further information on the LRRK2 cohort study, visit https://www.michaeljfox.org. The cohort comprised of 57 patients with idiopathic PD and 65 PD patients with the LRRK2 G2019S mutation. Patient samples were collected with informed consent and the research was approved by the University of Sydney Human Research Ethics Committee (2017/076 and 2017/857).

The proportion of the MJFF LRRK2 Cohort Consortium that was followed longitudinally was of Ashkenazi Jewish descent. The cohort had clinical data collected for PD participants at baseline (*N* = 122) and 2 years (*N* = 67). Demographic and clinical data accompanying the samples are available in Table 1. Clinical and demographic information regarding this cohort has been previously published^30^, although serum aliquot availability resulted in minor clinical/demographic variation in this study. The use of blood pressure, ibuprofen, aspirin and inflammatory medication were not available for the longitudinally followed proportion of the cohort. An exclusion criterion of a history of repeated head injury, definite encephalitis, cerebral tumour, MPTP exposure, stroke, epilepsy, inflammatory disease of the brain and skull fractures was applied. Subjects were fasted for a minimum of 8 h prior to sample donation with collections occurring between 8 and 10 am, or if fasting was not possible, subjects were to eat a low-lipid meal (see Supplementary File S1). *GBA1* genotypes were obtained from the MJFF-sponsored LRRK2 Cohort Consortium (accessed Feb 1^st^, 2024) with the aid of Dr Kelly Nudelman. Briefly, all known pathogenic *GBA1* variants (R535H, R502C, L483P, D448H, N409S, S212, R159W, 115 + 1 G > A and L29Afs*18) were checked from the Accelerating Medicines Partnership Parkinson’s disease (AMP-PD, https://amp-pd.org/) whole genome sequencing data set. To see if *GBA1* variants may have affected predictors, supplementary analyses were performed to compare the top predictors in Table 2 between *GBA1*+ (*n* = 6) and *GBA1-* (*n* = 42) cases with a 2 years follow-up and genotypes available.

### Untargeted serum lipidomics

Serum lipid levels were measured previously^28^, and the methods for obtaining this data are summarised in Fig. 1 and briefly repeated for convenience. Avanti lipid internal standards from twenty-one different classes were prepared and serum lipids were extracted via chloroform-methanol based on the method by Bligh and Dyer^46^. Lipid extracts (10 µl) were analysed by a blinded researcher using a Q-Exactive Plus Mass Spectrometer coupled to a U3000 UPLC system (Thermo Fisher Scientific). Chromatography was performed at 60;°C on a Waters CSH C18 ultra-high-performance liquid chromatography column 2.1 × 100 mm, 1.8 µM with VanGuard guard column. Solvent A was 6:4 acetonitrile:water and Solvent B was 1:9 acetonitrile:isopropanol, both with 10 mM ammonium formate and 0.1% formic acid. Lipids were chromatographed according to the method of ref. ^47^. Samples were run in both positive and negative polarities. MS data were searched against the standard LipidSearch database 4.2.23 with all common mammalian lipid classes included. Glycerolipids, glycerophospholipids, sterol lipids, fatty acyls, sphingolipids and prenol lipids were detected and 995 lipid species from 32 sub-classes (DG, MGDG, TG, BMP, LPC, LPE, PA, PC, PE, PEt, PG, PI, PS, Cer, CerG2NAc1, CerG3NAc1, CerPE, Hex1Cer, Hex2Cer, SM, phSM, ChE, AcHexChE, AcHexZyE, CmE, ZyE, AcCa, FA, OAHFA, CoQ8 and CoQ10) were analysed.

### Serum cytokines

The serum cytokine data used in this study was obtained from a previously described study and the methods for obtaining this data are repeated for convenience^30^. Briefly, a magnetic Bio-Plex Pro Human Cytokine 27-plex Assay (Biorad) was used by a blinded researcher to simultaneously measure 27 cytokines (MIP1β, IL-6, IFNγ, IL-1RA, IL-5, GM-CSF, TNFα, CCL5, IL-2, IL-1β, CCL11, bFGF, VEGF, PDGF, CXCL10, IL-13, IL-4, MCP-1, IL-8, MIP1α, IL-10, GCSF, IL-15, IL-7, IL-12(p70), IL-17A and IL-9) in the serum samples using a 1:4 dilution according to the manufacturer’s instructions. Plates were analysed using a Bio-Plex MAGPIX Multiplex plate reader (Bio-Rad).

### Neurofilament light and α-synuclein

Of the *N* = 67 PD participants which were followed up after 2 years, *N* = 64 baseline serum samples were available for both α-synuclein and neurofilament light (NfL) measurements. Sample order was randomised onto plates using the =RAND() function in Excel. Total serum α-synuclein was measured using the U-PLEX Human α-Synuclein Kit (#K151WKK-1, Mesoscale Discovery) according to the manufacturer instructions and as previously described^48^, using a 1:8 dilution of serum samples in duplicate. Controls provided with the U-Plex Plus kit (#K151WKP-2, Mesoscale Discovery) were additionally included. Eight calibrators were included in each plate and ranged from 0 to 10,000 pg/mL and were used to interpolate concentrations of total α-synuclein. The inter-assay CV% for the α-synuclein kit controls were ≤4.2%. The intra-assay CV% of samples was ≤11.3%. As haemolysis may affect measures, sera were visually inspected for haemolysis and two samples were excluded due to pink discoloration.

NfL was measured in serum using the R-PLEX Human Neurofilament L Assay (#K1517XR-2, Mesoscale Discovery), following Assay 1 of the manufacturer instructions and using the recommended 1:2 dilution of serum samples in duplicate. The eight calibrators included in each plate ranged from 0 - 50,000 pg/mL and were used to interpolate sample concentrations. The average intra-assay CV% of samples was 3.2% and all were ≤ 12.9% (two samples an intra-assay CV% of > 13% were excluded). Wash Buffer (#R61AA-1, Mesoscale Discovery) was used for U-Plex and R-Plex assays as per the kit manufacturer’s instructions.

### Statistical analysis

Data analysis was performed using SPSS (version 28) and R (version 4.2.3). Clinical variables across time points were assessed by one-way ANOVA with a least-significant difference post hoc test, except for Hoehn and Yahr, which was analysed via Kruskal Wallis and Mann-Whitney U tests. To ensure only markers with robust detection were included, lipid species that had >10% missing data were excluded from analyses. Missing data for the remaining lipid species were <1% of all lipids per case, with only a few exceptions. To prevent an entire case from being excluded from the models due to a small number of missing data points, means imputation was performed in cases with <1% to <10% missing lipid values. After splitting data into training (80%) and testing (20%) sets, the remaining lipid data had means imputation performed using the recipes package in R. Clinical data were not imputed. The lipidr package^49^ was used to perform orthogonal partial least-squares discriminant analysis (OPLS-DA), to generate a combination of baseline lipids which most separated the clinical outcomes after 2 years (referred to as ‘selected lipids’ from here on). The combinations of baseline predictors for each clinical outcome at 2 years were as follows: (1) baseline score of the predicted clinical scale + demographic data + all lipids + cytokines, (2) baseline score of the predicted clinical scale + demographic data + selected lipids + cytokines, (3) baseline score of the predicted clinical scale + demographic data + selected lipids, (4) selected lipids, (5) selected lipids + cytokines and (6) cytokines. Demographic data included age, sex and LRRK2 mutation status. The predictive performance of two machine learning models, random forest (1000 trees, V-fold cross-validation, *v* = 10 and repeats = 5) and elastic net linear regression (repeats = 5), were compared for all predictor combinations, using normalised route mean square error (NRMSE) and RMSE as a measure of performance. The hyperparameters for random forest (mtry) and elastic net (α and λ) which best minimised the RMSE were identified by 10-fold cross-validation on the training set. Both random forest and elastic net algorithms were split such that 80% of the samples were used for training and 20% were withheld for final validation. Stratified sampling of the testing and training dataset was performed for clinical data, to mitigate the risk of having imbalanced distributions of scores in either the training or testing set. An NRMSE score of zero indicates a prediction model of 100% accuracy. NfL and α-synuclein concentration values were log transformed to better fit a normal distribution and were subsequently added to the random forest models using selected lipids + baseline clinical scale + demographic data.

### Supplementary information

Supplementary Material

## Data Availability

The serum lipid and cytokine data and serum collection procedures are available upon request through the Michael J Fox Foundation (https://www.michaeljfox.org/news/lrrk2-cohort-consortium).

## Code availability

R code used in this study is available upon request.
