# Supplementary material for: Prediction of motor and non-motor Parkinson’s disease symptoms using serum lipidomics and machine learning: a 2-year study
Source: NPJ Parkinsons Dis. 2024 Jun 25;10:123. doi: 10.1038/s41531-024-00741-y (PMC11199659; doi:10.1038/s41531-024-00741-y)
Supplement: Supplementary file 1 — Supplementary Material [file 41531_2024_741_MOESM1_ESM.pdf]

File S1

Appendix P: Low-Fat Diet Menu Suggestions

PARKINSON'S PROGRESSION MARKERS INITIATIVE

Play a Part in Parkinson's Research

Low-Fat Diet Menu Suggestions

It is very important that you are fasted (no food and only drinks such as water, clear tea or black coffee) for at least 8 hours before your PPMI study visit for blood collection. However, we understand that fasting may not always be possible. If you are not able to fast, we ask that you choose from the suggested food items below. The PPMI study staff may provide additional suggestions for you.

Foods that are allowed prior to blood collection:

Sample Breakfast Items:

Dry whole-wheat toast
Fruit salad – no dressing
Clear tea or coffee (no milk or cream)
Fruit or vegetable juice

Dry cereal – (without nuts/ no granola; no milk)
Clear tea or coffee (no milk or cream)
Fruit or vegetable juice

Plain oatmeal or other cooked whole grain cereal
Topped with fresh or dried fruit (no butter, milk or cream)
Clear tea or coffee (no milk or cream)
Fruit or vegetable juice

Dry whole-wheat toast
Poached egg-whites or egg substitute
Clear tea or coffee (no milk or cream)
Fruit or vegetable juice

Sample Lunch Items:

Turkey breast sandwich on whole wheat bread
Lettuce and Tomato and Mustard
Clear beverage
Flavored gelatin

Plain pasta with plain marinara sauce– no butter or cheese
Side of steamed vegetables or green salad
Clear beverage
Flavored gelatin

Steamed chicken breast (lean, without skin)
Side of steamed vegetables or green salad
Clear beverage
Flavored gelatin

Large tossed green salad with assorted vegetables (no dressing or cheese)
Clear beverage
Flavored gelatin

Cucumber sandwich on whole-wheat bread
Lettuce, tomatoes, shredded carrots, onions or other vegetables
Clear beverage
Flavored gelatin

Clear broth with vegetables and pasta
Fruit salad – no dressing
Clear beverage
Flavored gelatin

v. 7/14/11

Foods to avoid prior to blood collection:

Avoid: All fats and nuts such as:

- Butter
- Cream
- Bacon fat
- Lard
- All oils
- All margarine
- All nuts
- Peanut butter
- Coconut
- Whole seeds such as pumpkin and sunflower

Avoid: All milk and dairy products such as:

- All whole milk products
- All cheeses
- All products containing cheese
- Cheeses spreads such as cream cheese
- Sour cream
- All ice cream
- Milk chocolate

Avoid: High fat prepared foods and foods naturally high in fat:

- All red meats or meats containing fat such as pork
- Fatty meats such as:
  - Luncheon meats
  - Organ meats
  - Bacon
- Fatty fish such as:
  - Salmon
  - Mackerel
- Salad dressing and mayonnaise
- Buttered, au gratin, creamed or fried vegetables.
- Fried foods
- Fried snacks such as:
  - Chips
  - Crackers
  - French fries
- Gravies and sauces
- Baked goods & frosting

Table S2

Model performance ***without*** *NfL and a-syn:*

Metric	Geriatric Depression	UPSIT	Schwab and England	UPDRS III
**RMSE**	2.6	6.13	14.1	11.91
**NRMSE**	0.72	0.76	0.93	0.73

Model performance ***with*** *NfL and a-syn:*

Metric	Geriatric Depression	UPSIT	Schwab and England	UPDRS III
**RMSE**	2.61	6.25	14.79	12.49
**NRMSE**	0.73	0.78	0.98	0.76

Table S3

Models with lowest NRMSE/RMSE from training set (variable importance list are of testing set)

GDS	Selected lipids + clinical data	
	Variable	Importance
1	GeriatricDepression_baseline	293.9
2	LPE 16:0	44.5
3	PE 18:1_20:4	35.1
4	SM d39:2	12.5
5	LPE 20:3	10.9
6	SM d40:3	10.8
7	LPC 16:0	7.5
8	PC 33:3	7.5
9	PAF 18:2e	7.0
10	PC 40:6	7.0
11	TG 16:0_14:0_18:3	6.9
12	LPC 22:5	6.2
13	PE 16:0_18:1	5.9
14	PE 18:0_18:1	5.9
15	PE 18:0_22:5	5.8
16	Age	5.6
17	PC 18:1_18:1	5.4
18	DG 20:0_18:1	5.4
19	LPC 20:4	5.1
20	DG 18:1_20:2	5.1
21	PC 35:2	4.9
22	DG 17:0_18:1	4.8
23	PC 32:1e	4.7
24	SM d36:1	4.7
25	TG 16:0_12:0_16:0	4.6
26	ChE 22:6	4.5
27	PC 37:6	4.2
28	DG 18:1_14:0	4.1
29	SM d38:2	4.1
30	TG 14:0_18:2_18:3	3.9
31	PC 44:6e	3.9
32	PC 36:2	3.9
33	PC 39:6	3.8
34	SM d44:5	3.8
35	DG 15:0_18:1	3.6
36	SM d18:1_24:2	3.6
37	PC 40:7	3.5
38	LPE 18:2	3.5
39	PC 32:0e	3.3
40	DG 18:1_18:1	3.3
41	PC 36:5e	3.2
42	LPE 18:1	3.1
43	SM d43:2	3.0
44	PC 38:6e	2.8
45	PC 16:1e_18:1	2.7
46	CerG2GNAc1 d36:3	2.6
47	PAF 12:0e	2.5
48	LPE 22:5	2.5
49	DG 16:0_18:1	2.5
50	DG 16:0_16:0	2.5
51	SM d40:0	2.5
52	DG 18:1_20:4	2.4
53	ChE 20:4	2.4
54	DG 20:1_18:1	2.4
55	DG 18:1_18:2	2.2
56	PC 34:1e	2.2
57	PC 33:1	2.1
58	SM d35:2	2.1
59	DG 20:3_18:2	2.1
60	SM t40:6	2.0
61	DG 20:0_18:2	2.0
62	TG 16:0_12:0_18:3	1.9
63	ChE 18:1	1.9
64	TG 16:0_12:0_18:2	1.9
65	TG 16:1_16:1_18:2	1.8
66	SM d43:5	1.7
67	DG 18:1_22:4	1.7
68	LPE 18:0	1.7
69	TG 16:1_18:2_18:3	1.6
70	PE 36:6e	1.5
71	DG 16:1_18:2	1.5
72	PC 40:7e	1.4
73	SM d42:4	1.4
74	SM d34:1	1.4
75	DG 18:1_20:3	1.4
76	DG 18:1_22:5	1.3
77	DG 18:0_18:1	1.3
78	PC 36:2e	1.3
79	SM d37:1	1.3
80	SM d18:1_17:0	1.2
81	DG 16:0_18:3	1.2
82	DG 17:1_18:1	1.2
83	DG 16:1_18:1	1.1
84	PC 40:6e	1.1
85	ChE 18:2	0.9
86	mutation_yes	0.9
87	SM d41:3	0.8
88	SM d18:1_24:1	0.7
89	gender_male	0.2

H&Y	Selected lipids + clinical data	
	Variable	Importance
1	HoehnandYhar_baseline	11.7
2	PC 32:1e	8.3
3	PAF 18:2e	2.4
4	PAF 12:0e	1.8
5	PC 35:2	1.2
6	CerG2GNAc1 d36:3	1.1
7	TG 16:0_12:0_16:0	0.9
8	LPC 20:4	0.8
9	PE 18:0_18:1	0.8
10	PE 18:1_20:4	0.7
11	SM d35:2	0.7
12	DG 15:0_18:1	0.6
13	Age	0.6
14	TG 16:1_18:2_18:3	0.6
15	PE 36:6e	0.6
16	LPE 20:3	0.6
17	LPE 18:2	0.5
18	PC 39:6	0.5
19	PE 16:0_18:1	0.5
20	PC 37:6	0.5
21	LPE 16:0	0.5
22	DG 16:0_16:0	0.4
23	PC 18:1_18:1	0.4
24	PC 33:1	0.4
25	DG 20:3_18:2	0.4
26	SM d40:0	0.4
27	LPE 18:1	0.4
28	PC 36:5e	0.3
29	SM d42:4	0.3
30	LPC 16:0	0.3
31	PC 40:6	0.3
32	PC 38:6e	0.3
33	PC 36:2	0.3
34	TG 14:0_18:2_18:3	0.3
35	TG 16:1_16:1_18:2	0.3
36	DG 18:1_20:4	0.3
37	SM d38:2	0.3
38	DG 18:1_20:3	0.3
39	DG 18:1_20:2	0.3
40	ChE 20:4	0.2
41	SM d18:1_24:2	0.2
42	TG 16:0_12:0_18:3	0.2
43	TG 16:0_14:0_18:3	0.2
44	SM d18:1_17:0	0.2
45	SM d36:1	0.2
46	DG 17:0_18:1	0.2
47	PC 33:3	0.2
48	LPE 18:0	0.2
49	TG 16:0_12:0_18:2	0.2
50	PC 40:7e	0.2
51	SM d34:1	0.2
52	DG 20:0_18:1	0.2
53	PC 40:7	0.2
54	PE 18:0_22:5	0.2
55	DG 20:0_18:2	0.2
56	DG 18:1_18:1	0.2
57	SM t40:6	0.2
58	SM d39:2	0.2
59	SM d44:5	0.2
60	DG 18:1_22:5	0.2
61	PC 44:6e	0.1
62	PC 40:6e	0.1
63	ChE 18:2	0.1
64	LPC 22:5	0.1
65	LPE 22:5	0.1
66	SM d18:1_24:1	0.1
67	SM d41:3	0.1
68	DG 16:1_18:2	0.1
69	SM d43:2	0.1
70	DG 17:1_18:1	0.1
71	ChE 22:6	0.1
72	PC 16:1e_18:1	0.1
73	SM d40:3	0.1
74	DG 18:0_18:1	0.1
75	DG 16:0_18:3	0.1
76	DG 20:1_18:1	0.1
77	PC 32:0e	0.1
78	SM d43:5	0.1
79	DG 18:1_14:0	0.1
80	ChE 18:1	0.1
81	DG 18:1_18:2	0.1
82	PC 36:2e	0.1
83	PC 34:1e	0.1
84	DG 16:1_18:1	0.1
85	SM d37:1	0.1
86	DG 18:1_22:4	0.1
87	DG 16:0_18:1	0.0
88	mutation_yes	0.0
89	gender_male	0.0

S&E	Selected lipids + clinical data	
	Variable	Importance
1	SchwabEngland_baseline	5846.4
2	PC 32:1e	1764.1
3	Age	1321.7
4	LPE 16:0	773.0
5	PE 16:0_18:1	634.7
6	PAF 12:0e	596.9
7	DG 16:0_18:3	562.7
8	PAF 18:2e	463.3
9	PE 18:1_20:4	388.7
10	PE 18:0_18:1	374.1
11	DG 18:1_20:3	373.9
12	PC 36:5e	342.6
13	PC 35:2	305.7
14	DG 15:0_18:1	282.7
15	LPE 20:3	281.3
16	TG 16:0_12:0_16:0	217.7
17	TG 16:0_12:0_18:3	210.0
18	PC 40:6	193.3
19	PE 36:6e	180.4
20	LPE 18:2	177.6
21	PC 33:1	176.4
22	DG 20:3_18:2	167.9
23	PC 36:2	113.1
24	PC 18:1_18:1	108.0
25	SM d35:2	101.1
26	ChE 20:4	93.2
27	DG 18:1_20:4	92.1
28	DG 16:0_16:0	88.0
29	PC 39:6	85.9
30	DG 17:0_18:1	84.8
31	DG 20:0_18:1	79.8
32	PE 18:0_22:5	77.9
33	TG 16:1_18:2_18:3	74.8
34	ChE 18:1	73.5
35	PC 40:6e	71.3
36	TG 16:1_16:1_18:2	70.6
37	SM d40:0	69.2
38	SM d39:2	69.2
39	LPE 18:1	69.1
40	SM t40:6	68.7
41	DG 16:1_18:1	68.3
42	TG 16:0_12:0_18:2	67.5
43	ChE 22:6	66.4
44	TG 14:0_18:2_18:3	63.5
45	CerG2GNAc1 d36:3	59.9
46	PC 40:7	55.8
47	DG 18:1_22:5	54.6
48	DG 20:0_18:2	53.6
49	SM d42:4	51.7
50	DG 18:0_18:1	51.7
51	DG 18:1_20:2	51.6
52	PC 33:3	50.6
53	DG 16:0_18:1	49.7
54	DG 20:1_18:1	47.5
55	SM d43:5	47.3
56	SM d34:1	47.0
57	SM d44:5	46.9
58	SM d43:2	46.9
59	SM d38:2	46.8
60	PC 40:7e	45.5
61	PC 38:6e	43.1
62	PC 44:6e	42.3
63	TG 16:0_14:0_18:3	41.6
64	SM d18:1_24:1	41.6
65	LPE 18:0	41.2
66	SM d18:1_24:2	40.1
67	SM d18:1_17:0	39.7
68	SM d36:1	39.2
69	LPE 22:5	38.6
70	DG 18:1_14:0	37.8
71	SM d41:3	36.0
72	PC 37:6	35.8
73	PC 36:2e	34.7
74	ChE 18:2	34.6
75	DG 16:1_18:2	32.1
76	DG 17:1_18:1	32.0
77	SM d37:1	29.7
78	DG 18:1_22:4	29.6
79	LPC 20:4	29.4
80	LPC 22:5	28.3
81	SM d40:3	28.0
82	DG 18:1_18:2	24.7
83	LPC 16:0	24.3
84	DG 18:1_18:1	24.0
85	PC 16:1e_18:1	23.7
86	PC 32:0e	23.4
87	PC 34:1e	16.4
88	gender_male	6.8
89	mutation_yes	1.7

UPSIT	Selected lipids + clinical data	
	Variable	Importance
1	UPSIT_baseline	1459.2
2	PAF 12:0e	86.7
3	PC 39:6	83.3
4	PC 38:6e	38.3
5	PC 40:6	31.1
6	LPC 20:4	23.7
7	PC 37:6	22.9
8	TG 14:0_18:2_18:3	21.3
9	PC 44:6e	18.3
10	DG 18:1_18:2	18.3
11	PC 32:1e	17.9
12	ChE 20:4	17.8
13	PC 33:1	17.5
14	TG 16:0_12:0_16:0	15.5
15	DG 20:0_18:1	14.6
16	TG 16:0_12:0_18:2	14.5
17	SM d18:1_17:0	13.6
18	PC 40:7	12.9
19	SM d43:2	12.5
20	PAF 18:2e	12.1
21	LPE 18:2	12.0
22	LPC 22:5	11.6
23	TG 16:1_18:2_18:3	10.5
24	DG 17:0_18:1	10.0
25	SM d37:1	9.6
26	DG 18:1_14:0	9.5
27	Age	9.3
28	SM t40:6	8.8
29	LPC 16:0	8.8
30	TG 16:0_14:0_18:3	8.6
31	LPE 20:3	8.5
32	PC 33:3	8.4
33	PE 18:0_18:1	8.2
34	TG 16:0_12:0_18:3	7.4
35	DG 20:1_18:1	7.2
36	SM d38:2	7.0
37	LPE 18:1	6.7
38	ChE 22:6	6.7
39	LPE 22:5	6.6
40	PC 36:5e	6.3
41	SM d42:4	6.3
42	DG 16:0_16:0	6.3
43	SM d40:0	6.2
44	SM d36:1	6.1
45	DG 20:3_18:2	5.9
46	SM d39:2	5.9
47	DG 18:1_18:1	5.5
48	PE 16:0_18:1	5.4
49	PC 18:1_18:1	5.3
50	PC 36:2	5.2
51	DG 18:1_20:2	5.1
52	CerG2GNAc1 d36:3	4.9
53	DG 18:1_20:4	4.5
54	PC 35:2	4.5
55	SM d35:2	4.1
56	SM d18:1_24:2	4.1
57	PC 40:6e	4.0
58	TG 16:1_16:1_18:2	3.9
59	PE 18:1_20:4	3.9
60	SM d40:3	3.9
61	PE 18:0_22:5	3.8
62	SM d44:5	3.8
63	ChE 18:2	3.6
64	DG 16:1_18:1	3.6
65	ChE 18:1	3.6
66	DG 16:0_18:3	3.5
67	SM d34:1	3.5
68	SM d18:1_24:1	3.4
69	PC 40:7e	3.4
70	DG 15:0_18:1	3.4
71	DG 20:0_18:2	3.3
72	PE 36:6e	3.3
73	LPE 18:0	3.1
74	LPE 16:0	3.0
75	mutation_yes	3.0
76	DG 16:0_18:1	3.0
77	DG 18:1_20:3	2.8
78	DG 18:0_18:1	2.7
79	DG 18:1_22:4	2.6
80	DG 18:1_22:5	2.5
81	PC 32:0e	2.5
82	PC 34:1e	2.5
83	SM d43:5	2.4
84	SM d41:3	2.3
85	DG 16:1_18:2	2.3
86	DG 17:1_18:1	2.1
87	PC 16:1e_18:1	2.1
88	PC 36:2e	1.7
89	gender_male	0.2

UPDRSIII	Selected lipids + clinical data	
	Variable	Importance
1	UPDRSIII_baseline	3319.1
2	Age	190.0
3	TG 14:0_18:2_18:3	164.1
4	PAF 12:0e	163.0
5	PC 33:3	144.6
6	DG 20:3_18:2	141.5
7	LPE 20:3	134.1
8	SM d18:1_17:0	125.5
9	PC 32:0e	119.8
10	PC 44:6e	118.2
11	PAF 18:2e	106.0
12	PC 32:1e	105.9
13	TG 16:1_16:1_18:2	80.8
14	LPE 16:0	80.0
15	DG 18:1_20:3	79.0
16	PC 36:2	67.6
17	DG 20:0_18:2	67.0
18	TG 16:0_12:0_16:0	65.6
19	PE 16:0_18:1	64.0
20	PC 36:5e	62.8
21	TG 16:1_18:2_18:3	60.2
22	SM d43:2	60.0
23	DG 15:0_18:1	55.7
24	SM d38:2	51.8
25	DG 18:1_18:2	50.6
26	PC 40:6e	47.7
27	LPE 18:1	47.4
28	TG 16:0_14:0_18:3	46.7
29	ChE 22:6	45.4
30	LPE 18:2	44.6
31	PE 18:0_22:5	43.7
32	PC 37:6	43.4
33	SM d36:1	42.5
34	SM d39:2	42.4
35	SM d44:5	42.2
36	DG 20:1_18:1	41.8
37	PC 38:6e	38.5
38	DG 17:0_18:1	37.0
39	ChE 20:4	36.9
40	PC 33:1	36.2
41	DG 16:0_16:0	36.2
42	PC 40:7	34.6
43	LPC 22:5	34.0
44	DG 20:0_18:1	32.1
45	SM d42:4	31.1
46	PC 18:1_18:1	30.7
47	PE 18:1_20:4	30.3
48	CerG2GNAc1 d36:3	28.7
49	SM d40:0	28.7
50	PE 36:6e	27.7
51	DG 16:1_18:2	27.2
52	LPC 16:0	25.1
53	PC 39:6	25.0
54	SM d37:1	24.4
55	LPC 20:4	24.0
56	SM d35:2	23.5
57	DG 18:1_20:2	23.2
58	PC 35:2	22.7
59	DG 18:1_20:4	22.3
60	PC 16:1e_18:1	22.2
61	DG 18:1_14:0	21.9
62	DG 16:0_18:3	21.7
63	TG 16:0_12:0_18:3	20.9
64	TG 16:0_12:0_18:2	19.7
65	LPE 22:5	19.7
66	DG 18:0_18:1	18.6
67	SM d41:3	18.4
68	SM t40:6	17.3
69	LPE 18:0	16.8
70	ChE 18:2	16.7
71	SM d34:1	16.7
72	SM d18:1_24:1	16.5
73	PC 34:1e	16.0
74	DG 18:1_22:5	15.7
75	PC 40:6	15.7
76	SM d43:5	15.3
77	ChE 18:1	13.7
78	PC 40:7e	13.3
79	PC 36:2e	12.1
80	PE 18:0_18:1	12.0
81	DG 16:1_18:1	11.8
82	SM d18:1_24:2	11.7
83	DG 17:1_18:1	11.2
84	SM d40:3	11.1
85	DG 16:0_18:1	10.3
86	DG 18:1_22:4	9.4
87	gender_male	6.4
88	DG 18:1_18:1	6.3
89	mutation_yes	3.4

Table S4

Correlations

LED 2 years					
		**LPE 16:0**	**PE 18:1_20:4**	**SM d39:2**	**LPE 20:3**
	**Correlation**	0.473	-0.321	0.445	-0.016
	**Significance (2-tailed)**	0.343	0.535	0.376	0.976
	**df**	4	4	4	4
		**SM d40:3**	**LPC 16:0**	**PC 33:3**	**PAF 18:2e**
	**Correlation**	0.358	0.171	0.578	0.151
	**Significance (2-tailed)**	0.486	0.746	0.23	0.775
	**df**	4	4	4	4
		**PC 40:6**	**PC 32:1e**	**LPE 16:0**	**PE 16:0_18:1**
	**Correlation**	-0.096	0.784	0.473	-0.08
	**Significance (2-tailed)**	0.856	0.065	0.343	0.88
	**df**	4	4	4	4
		**PAF 12:0e**	**DG 16:0_18:3**	**PAF 18:2e**	**PE 18:1_20:4**
	**Correlation**	0.235	-0.362	0.151	-0.321
	**Significance (2-tailed)**	0.653	0.48	0.775	0.535
	**df**	4	4	4	4
		**PE 18:0_18:1**	**PAF 12:0e**	**PC 39:6**	**PC 38:6e**
	**Correlation**	0.479	0.235	-0.03	0.035
	**Significance (2-tailed)**	0.336	0.653	0.956	0.948
	**df**	4	4	4	4
		**PC 40:6**	**LPC 20:4**	**PC 37:6**	**TG 14:0_18:2_18:3**
	**Correlation**	-0.096	-0.01	0.048	-0.054
	**Significance (2-tailed)**	0.856	0.986	0.928	0.919
	**df**	4	4	4	4
		**PC 44:6e**	**DG 18:1_18:2**	**TG 14:0_18:2_18:3**	**PAF 12:0e**
	**Correlation**	0.28	-0.476	-0.054	0.235
	**Significance (2-tailed)**	0.591	0.34	0.919	0.653
	**df**	4	4	4	4
		**PC 33:3**	**DG 20:3_18:2**	**LPE 20:3**	**SM d18:1_17:0**
	**Correlation**	0.578	-0.519	-0.016	-0.39
	**Significance (2-tailed)**	0.23	0.291	0.976	0.445
	**df**	4	4	4	4
		**PC 32:0e**	**PC 44:6e**		
	**Correlation**	0.339	0.28		
	**Significance (2-tailed)**	0.511	0.591		
	**df**	4	4		

Table S5

	GBA1-	GBA1+	*p* -value
LPE 16:0	2.032 ± 0.046	2.033 ± 0.125	$p > 0.05$
PE 18:1_20:4	1.456 ± 0.071	1.563 ± 0.193	$p > 0.05$
SM d39:2	1.225 ± 0.032	1.255 ± 0.086	$p > 0.05$
LPE 20:3	0.827 ± 0.029	0.891 ± 0.078	$p > 0.05$
SM d40:3	0.878 ± 0.020	0.941 ± 0.054	$p > 0.05$
LPC 16:0	19.616 ± 0.424	18.501 ± 1.149	$p > 0.05$
PC 33:3	0.875 ± 0.040	0.94 ± 0.108	$p > 0.05$
PAF 18:2e	1.016 ± 0.031	1.064 ± 0.085	$p > 0.05$
PC 40:6	5.636 ± 0.145	5.695 ± 0.394	$p > 0.05$
PC 32:1e	1.353 ± 0.48	1.32 ± 0.129	$p > 0.05$
PE 16:0_18:1	1.237 ± 0.044	1.155 ± 0.120	$p > 0.05$
PAF 12:0e	2.123 ± 0.082	1.940 ± 0.222	$p > 0.05$
DG 16:0_18:3	3.574 ± 0.194	3.511 ± 0.525	$p > 0.05$
PE 18:0_18:1	1.666 ± 0.057	1.582 ± 0.154	$p > 0.05$
PC 39:6	1.188 ± 0.042	1.332 ± 0.113	$p > 0.05$
PC 38:6e	1.435 ± 0.050	1.579 ± 0.134	$p > 0.05$
LPC 20:4	3.507 ± 0.122	3.611 ± 0.330	$p > 0.05$
PC 37:6	1.394 ± 0.051	1.397 ± 0.140	$p > 0.05$
TG 14:0_18:2_18:3	0.733 ± 0.035	0.786 ± 0.095	$p > 0.05$
PC 44:6e	1.551 ± 0.047	1.606 ± 0.127	$p > 0.05$
DG 18:1_18:2	25.301 ± 1.122	25.162 ± 3.043	$p > 0.05$
DG 20:3_18:2	2.471 ± 0.131	2.583 ± 0.354	$p > 0.05$
SM d18:1_17:0	1.018 ± 0.029	1.121 ± 0.079	$p > 0.05$
PC 32:0e	1.662 ± 0.045	1.780 ± 0.123	$p > 0.05$

Vales represent mean of normalised lipid ± SEM
